# Evidence- and data-driven classification of low back pain via artificial intelligence: Protocol of the PREDICT-LBP study

**DOI:** 10.1371/journal.pone.0282346

**Published:** 2023-08-21

**Authors:** Daniel L. Belavy, Scott D. Tagliaferri, Martin Tegenthoff, Elena Enax-Krumova, Lara Schlaffke, Björn Bühring, Tobias L. Schulte, Sein Schmidt, Hans-Joachim Wilke, Maia Angelova, Guy Trudel, Katja Ehrenbrusthoff, Bernadette Fitzgibbon, Jessica Van Oosterwijck, Clint T. Miller, Patrick J. Owen, Steven Bowe, Rebekka Döding, Svenja Kaczorowski

**Affiliations:** 1 Division of Physiotherapy, Department of Applied Health Sciences, Hochschule für Gesundheit (University of Applied Sciences), Bochum, Germany; 2 Institute for Physical Activity and Nutrition, School of Exercise and Nutrition Sciences, Deakin University, Geelong, Victoria, Australia; 3 Department of Neurology, BG-University Hospital Bergmannsheil gGmbH, Ruhr-University Bochum, Bochum, Germany; 4 Internistische Rheumatologie, Krankenhaus St. Josef Wuppertal, Wuppertal, Germany; 5 Department of Orthopaedics and Trauma Surgery, St. Josef-Hospital Bochum, Ruhr University Bochum, Bochum, Germany; 6 Berlin Institute of Health, Charité Universitätsmedizin Berlin, Berlin, Germany; 7 Institute of Orthopaedic Research and Biomechanics, Trauma Research Center Ulm, University Hospital Ulm, Ulm, Germany; 8 School of Information Technology, Deakin University, Geelong, Australia; 9 Clinical Epidemiology Program, Ottawa Hospital Research Institute, Ottawa, Canada; 10 Monarch Research Institute, Monarch Mental Health Group, Melbourne, Australia; 11 School of Psychology and Medicine, Australian National University, Canberra, Australia; 12 Department of Psychiatry, Monash University, Melbourne, Australia; 13 Faculty of Medicine and Health Sciences, Ghent University, Ghent, Belgium; 14 Faculty of Health, Deakin University, Geelong, Australia; 15 Te Kura Tātai Hauora-The School of Health, Victoria University of Wellington, Wellington, New Zealand; PLOS: Public Library of Science, UNITED KINGDOM

## Abstract

In patients presenting with low back pain (LBP), once specific causes are excluded (fracture, infection, inflammatory arthritis, cancer, cauda equina and radiculopathy) many clinicians pose a diagnosis of non-specific LBP. Accordingly, current management of non-specific LBP is generic. There is a need for a classification of non-specific LBP that is both data- and evidence-based assessing multi-dimensional pain-related factors in a large sample size. The “PRedictive Evidence Driven Intelligent Classification Tool for Low Back Pain” (PREDICT-LBP) project is a prospective cross-sectional study which will compare 300 women and men with non-specific LBP (aged 18–55 years) with 100 matched referents without a history of LBP. Participants will be recruited from the general public and local medical facilities. Data will be collected on spinal tissue (intervertebral disc composition and morphology, vertebral fat fraction and paraspinal muscle size and composition via magnetic resonance imaging [MRI]), central nervous system adaptation (pain thresholds, temporal summation of pain, brain resting state functional connectivity, structural connectivity and regional volumes via MRI), psychosocial factors (e.g. depression, anxiety) and other musculoskeletal pain symptoms. Dimensionality reduction, cluster validation and fuzzy c-means clustering methods, classification models, and relevant sensitivity analyses, will classify non-specific LBP patients into sub-groups. This project represents a first personalised diagnostic approach to non-specific LBP, with potential for widespread uptake in clinical practice. This project will provide evidence to support clinical trials assessing specific treatments approaches for potential subgroups of patients with non-specific LBP. The classification tool may lead to better patient outcomes and reduction in economic costs.

## Introduction

Back pain is the greatest cause of disability and lost productivity [1] and affects approximately 540 million people worldwide [2]. Reducing the burden of disease associated with low back pain (LBP) is a public health priority. Approximately 90% of LBP have been classified as non-specific [3]. A debate exists as to what is considered non-specific LBP [3–5]. Most authors agree that non-degenerative spinal diseases (e.g.; fracture, cancer, infection and inflammatory spondyloarthropathy) and damage to the spinal nervous system (e.g.; cauda equina, spinal stenosis and radiculopathy) are specific causes of LBP [3–5]. However, degenerative spinal conditions (e.g.; degenerative disc disease, facet arthropathy, spondylolysis) are considered specific by some authors [5]. Others argue they are non-specific [3, 4] based on evidence that clinical tests cannot reliably attribute pain to specific structures and that degenerative spine conditions can be present in people with or without LBP [6–9].

Recommendations for the treatment of non-specific LBP are by necessity generic when it is unclear what is contributing to the pain in each patient. These include exercise, manual therapy, psychological interventions, medication and multidisciplinary interventions [10]. The efficacy of these treatments on non-specific LBP often do not reach the threshold of clinical significance in many patients [3]. Clinical classification approaches exist for the conservative management of LBP, such as McKenzie treatment and STarT Back, but their impact is small and effect size not clinically meaningful versus more generic treatments [11]. A better clinical classification of LBP is a prerequisite to develop targeted treatment approaches.

Multiple domains impact a person experience of pain [12, 13]. In non-specific LBP, damage to the spinal tissues (nociceptive pain drivers), alterations in the processing of the pain signal (nervous system dysfunction), presence of other musculoskeletal diseases (comorbidity drivers), negative mood/affect, poor self-efficacy, and the use of maladaptive coping strategies (cognitive-emotional drivers) can influence the pain experience [6–9, 13]. Further, abnormal central pain processing including central sensitisation [14, 15], reorganisation of (sub)cortical brain pathways [16], and changes in brain connectivity [17–19] can contribute to pain. Beliefs, fear of movement, depressive symptoms, anxiety, past experiences, self-efficacy, presence of other painful conditions may mediate the non-specific LBP experience [20, 21].

Of the known factors which may contribute to an individual’s experience of non-specific LBP, there is not a single one that can differentiate between those who have non-specific LBP and those who are pain free [22]. In a recent systematic review with multivariate meta-analysis [23] we showed that multiple domains are impacted in LBP and that there was only one primary study [24], of small sample size (n = 32) which considered more than two domains of central nervous system adaptation, tissue changes and/or psychological factors.

Artificial intelligence (AI) and machine learning offer automated methods to detect unique sub-groups that ‘explain’ the presence and characteristics of a disease [25, 26]. AI techniques have been used in other diseases (e.g. severe sepsis [27], cardiovascular disease [28], diabetes [29]) to improve clinical outcomes. A systematic review of AI applications in LBP [30] noted that (a) no studies to date considered if possible sub-groups exist, (b) a limited number of body systems were evaluated and (c) sample sizes remained small (ranging from 24 to 171 participants in these studies). There is an evidence gap for a study to use data-science techniques in LBP in an appropriate sample size. Since this review, work from our group [31], using data from the UK Biobank, showed that sub-groups could be derived in (chronic) LBP on the basis of psychosocial variables and then accurately classified, but the data set lacked spinal tissue and pain sensitisation measures and key clinical data (e.g. pain intensity, disability and pain duration).

The key path forward is to prospectively collect data from multiple domains known to contribute to the experience LBP in both a LBP and pain-free collective. The application of AI and data science techniques can then be used to examine for the presence of sub-groups. The aim of the “PRedictive Evidence Driven Intelligent Classification Tool for Low Back Pain” (PREDICT-LBP) project is to develop a prospective, evidence- and data-driven tool for determining sub-groups of patients under the common label of non-specific LBP using clinical measures and machine learning algorithms.

## Materials and methods

### Study design

This prospective cross-sectional study will compare people with non-specific LBP, as defined in prior work [3], with age-, sex- and BMI-matched referents without a history of LBP. The study was pre-registered at the German Clinical Trial Register (DRKS00030518). The protocol is in accordance with the Helsinki Declaration of ethical principles [32] and has been approved by the local ethical committee (*Ethik-Kommission der Hochschule für Gesundheit*; reference number 210827_Belavy). Written informed consent will be obtained from all participants. The STROBE guideline [33] and TRIPOD statement [34] for prediction model development were used to report the sections in this protocol (S1 and S2 Tables respectively). We will update our reporting to the TRIPOD-artificial intelligence statement [35] if it is released during our project.

### Setting

Participant recruitment will start in the beginning of 2023 and continue for 18 months. It will apply the use of a variety of recruitment procedures (i.e. postal flyers, notice boards, internet approaches, social media) at the Hochschule für Gesundheit Bochum [University of Applied Sciences], in the general public (i.e. local companies, sports centres, ambulatory medical practices), local hospitals (Uniklinik Bergmannsheil Bochum, St. Josef-Hospital Uniklinik Bochum, St. Josef-Hospital Wuppertal) and general practitioners as well as specialist outpatient clinics in the community. Expanded recruitment strategies are effective in obtaining a more balanced sample and increasing the external validity of study results [36, 37]. Recruitment will be stratified equally in each of four age groups (18-25yr, 26-35yr, 36-45yr, 46-55yr), aiming for a 55:45 ratio of women to men due to higher prevalence of LBP in women [2]. Furthermore, the prevalence of acute versus chronic back pain in the wider community is unclear with wide confidence intervals [38]. Cohort studies [39] can inform on prognosis of people with back pain but not the proportion of the population suffering acute versus chronic back pain at any given time. We will therefore pragmatically recruit participants with LBP to target a median duration of pain in the final collective of between three and five months. If eligible and participants consent to participate, study procedures will be completed within six weeks of the date of consent; as some measures may reflect not only traits but also states. Participants will receive a payment of 100 EUR upon completion of measures.

Data collection will occur at the Hochschule für Gesundheit Bochum [University of Applied Sciences] (Tactile spatial acuity assessment, pressure pain thresholds, temporal summation of pain, laser evoked potentials, questionnaires, trunk muscle strength and endurance) and at the Uniklinik Bergmannsheil Bochum (magnetic resonance imaging [MRI] scanning). Personal data of participants will be pseudonymized between the two settings and transmitted via phone call or email.

### Participants

In defining the inclusion and exclusion criteria the following underlying principles will be used: firstly, people with specific causes of LBP will be excluded: a) non-degenerative spinal diseases (e.g.; fracture, cancer, infection and inflammatory spondyloarthropathy) and b) damage to the spinal nervous system (e.g.; cauda equina, spinal stenosis and radiculopathy). Secondly, we consider the debate about whether degenerative spinal conditions constitute specific or non-specific LBP as clinical equipoise justifying the need for further research. In this study, we will consider degenerative spinal conditions as non-specific while some severe deformity will be considered specific and excluded. Thirdly, as our aim is to develop a clinical categorisation approach for non-specific LBP that could be of utility in clinical practice, we aim to recruit a collective representative of LBP in the wider population. Fourthly, participants enrolled in the matched control group should have similar characteristics as people with LBP, only without LBP.

Inclusion criteria for both LBP and matched controls: adults aged 18 to 55 years, and being fully conversant in German or English. The age bracket 18 to 55 will be targeted as first incidence of LBP typically occurs before or during mid-life and the peak burden of disease is in the age bracket below 55yrs [2].

Inclusion criterion for LBP: pain localised below the costal margin and above the inferior gluteal folds, with or without leg pain [40].

Exclusion criteria for both LBP and matched controls will be:

- Known clinical diagnosis of non-degenerative specific spinal conditions: cancer, vertebral fracture, vertebral or intervertebral disc infection or inflammatory spondyloarthropathy.
- Known clinical diagnosis of spinal nervous system damage: cauda equina, spinal stenosis [41] or diagnosed radiculopathy [42] as defined in prior work. Leg/radiating pain is not an exclusion criterion, nor is spinal stenosis or foraminal stenosis on imaging with no neurological damage.
- Known clinical diagnosis of severe degenerative spinal conditions: grade IV spondylolisthesis, severe deformity (e.g. hemivertebrae, scoliosis > 50 degrees) requiring more than two prior medical specialist consultations.
- Previous spine surgery (being on a waiting list for surgery is, of itself, not an exclusion).
- Prior major head trauma or brain surgery.
- Known clinical diagnoses of the following neurological conditions: cerebral palsy, dementia, epilepsy, motor neurone disease, multiple sclerosis, Parkinson’s disease, spinal muscular atrophy, stroke, polyneuropathy.
- Known major psychiatric disorder: mania, bipolar affective disorder, major depressive disorder, severe personality disorder, severe anxiety disorder, severe obsessive-compulsive disorder [43] as these may impact brain MRI measures [44].
- Current pregnancy or up to 6 months post-pregnancy (pelvic girdle pain associated with pregnancy typically resolves in ~80% of women by 6 months post-partum [45, 46]).
- Any further absolute contraindications for brain or spine MRI (e.g. cardiac pacemakers, metal implants and foreign bodies, middle ear implants, drug pumps, electronic stimulation devices).

For the pain-free control group, the following additional exclusion criteria will be applied:

- current spinal (neck, upper back, or low back) pain impacting function (self-report),
- LBP lasting for more than 24 hours within the last year (except for muscle soreness related to physical activity or exercise),
- previously, at any point, missed days from work due to LBP,
- previously, at any time, consulted a health professional for the treatment of LBP (e.g. physiotherapist and general practitioner).

Pain-free controls will be matched to LBP participants on a 1:3 basis (see Sample Size section for rationale) by

- sex (male or female)
- age brackets (18-25yr, 26–3, 36-45yr, 46-55yr)
- height (±5 cm; as body height has an impact on spine morphology)
- additionally, where possible, a body mass index of ±5 kg/m^2^ will be preferred.

Method of ascertainment: a screening tool will be used to assess the eligibility of the participants for the study. The screening tool will be implemented by study staff (e.g. study nurse, project manager) as part of verbal screening of the potential candidates for participation.

### Quantitative variables and data collection

Each participant will undergo an integrated test battery, which will assess nervous system adaptations, cognitive-emotional factors, co-morbid musculoskeletal disease, and standard outcome measures to describe the collective. Table 1 presents variables that flow into data-analytics versus those that are considered in secondary analyses only as it is less clear whether these latter variables can be “causal” for LBP.

**Table 1:** Variables to be assessed in analytical methods.

Variable	Specification	Unit	Type (range)
Lumbar spine MRI			
Intervertebral disc T2	Average L1/2-L5/S1 (from central three slices of T2-spin echo multi echo sequence)	ms	Continuous (0→)
Intervertebral disc height	Average L1/2-L5/S1 (from central three slices of T2-spin echo multi echo sequence)	mm	Continuous (0→)
Intervertebral disc volume	Average L1/2-L5/S1 (from all slices of T2-spin echo multi echo sequence)	cm^3^	Continuous (0→)
Lumbar vertebra fat fraction	Average L1-L5 (Sagittal DIXON sequence)	%	Continuous (0–100%)
Paraspinal muscle size	Volume of the multifidus, erector spinae, psoas major and quadratus lumborum muscles (averaged for left and right sides; Axial DIXON sequence)	cm^3^	Continuous (0→)
Paraspinal muscle composition	Fat fraction of the multifidus, erector spinae, psoas major and quadratus lumborum muscles (averaged for left and right sides; Axial DIXON sequence)	%	Continuous (0–100%)
Radiographic grading	Average Pfirrmann grading across all lumbar levels (1–5); Average facet joint grading across lumbar levels (0–3); Average pars grading across all lumbar levels (0–4); Average disc bulge grade across all lumbar levels (0–3)	NA	Continuous (ordinal values averaged between multiple levels)
Brain MRI			
Resting state functional connectivity	Connectivity within and between the default mode, frontoparietal, visual and sensorimotor networks.	z	Continuous (←0→)
Structural connectivity between pain related networks	Structural connectivity in pain networks using diffusion based tractography, regional alterations in white matter structure using ROI analysis. Outcome measure: Fractional anisotropy; Mean, radial and axial diffusion; number of fiber tracts between pre-defined regions of interest	unitless; count; mm^2^/s	Continuous (0→1); Continuous (0→); Continuous (0→)
Grey matter volumes	Medial frontal cortex, amygdala, thalamus, insula, caudate, putamen, anterior cingulate cortex, hippocampus, precentral gyrus (primary motor cortex), supplementary motor cortex, post-central gyrus (primary somatosensory cortex) and parietal operculum (secondary somatosensory cortex).	mm^3^	Continuous (0→)
Tactile spatial acuity assessment			
Two-point discrimination threshold	Lumbar region (minimum distance)	mm	Continuous (0→)
Pressure pain thresholds			
Local hyperalgesia	Lumbar paraspinal muscles (averaged across left and right sides)	kPA	Continuous (0→)
Widespread hyperalgesia	Average pain threshold from elbow and calf (averaged across left and right sides and sites)	kPA	Continuous (0→)
Temporal summation of pain			
Temporal summation (change in verbal NRS)	Lumbar paraspinal muscles, elbow and calf (averaged across left and right sides and sites)	NA	Continuous (0–10)
Laser evoked potentials			
Laser evoked potentials	Latencies of cortical N1, N2, P2 components	ms	Continuous (0→)
	Amplitudes of cortical N2-P2 complex recorded at CZ	μV	Continuous (0→)
Questionnaire: Cognitive-emotional factors			
PROMIS	Anxiety	NA	Continuous (8–40; lower scores are better)
PROMIS	Depression	NA	Continuous (8–40; lower scores are better)
PROMIS	Cognitive function	NA	Continuous (8–40; higher scores are better)
PROMIS	Self-efficacy	NA	Continuous (10–50; higher scores are better)
PROMIS	Satisfaction in social roles and activities	NA	Continuous (8–40; higher scores are better)
PROMIS	Social isolation	NA	Continuous (8–40; higher scores are better)
Perceived Social Support Questionnaire (F-SozU)	Social and instrumental support	NA	Continuous (5–30)
Questionnaire: Central sensitization			
Central Sensitisation Inventory	Total score	NA	Continuous (0–100)
Variables for consideration only in secondary analyses			
Gender (female, male)	(female = 0, male = 1)	NA	Categorical
Body mass index		kg/m^2^	Continuous (0→)
Age		yr	Continuous (18–55)
Pain duration	Acute (0-6wk), sub-acute (6-12wk), chronic (12wk+)	wk	Categorical
Pain intensity	Mild-moderate (VAS <50mm) vs moderate-severe (VAS>50mm)	mm	Continuous (0→100)
Nordic musculoskeletal questionnaire	Number of pain sites over the last 12 months aside from the lower back	NA	Continuous (0–9)
Smoking	Current, prior, never	NA	Categorical
Cardiovascular risk factors	known diabetes, hypertension, high cholesterol and parent who had suffered a myocardial infarction before age 60	NA	Categorical (yes/no)
Occupation	Classified according to physical activity characteristics	NA	Categorical
Trunk muscle endurance	Maximal trunk flexion and extension endurance	s	Continuous (0→)
Trunk muscle strength	Maximal trunk extension strength	kg	Continuous (0→)

NRS: numerical rating scale. PROMIS: Patient-Reported Outcomes Measurement Information System. F-SozU: Brief form of the Perceived Social Support Questionnaire (F-SozU K-6). VAS: visual analogue scale for pain

#### Brain MRI

A 3T MRI scanner with a 32 channel receive-only phased-array head coil will be used. As in preliminary work [31, 47], high resolution magnetisation prepared rapid acquisition gradient echo (MP2RAGE) T1-weighted structural data will be acquired to assess grey matter volumes. Resting state functional connectivity in pain networks will be assessed using an EPI sequence and those data will be pre-processed, denoised and analysed using standardized pipelines. Fieldmaps will also be attained in the scanning pipeline to allow for resting state MRI distortion correction. Diffusion weighted images will be obtained using a EPI-sequences with 60 isotropic oriented gradient orientations and various b-values. These data will be processed to assess regional white matter alterations in pain related brain areas. Furthermore, tractography will be performed to analyse structural connectivity within pain networks and to correlate the structural connectivity to functional connectivity obtained by resting state fMRI.

Contention remains regarding the most important functional networks and brain regions that contribute to non-specific LBP. Therefore, we will assess a wide range of variables (Table 1) and prior to deciding which variables that go to further data processing, dimensionality reduction will performed (see Analytical Methods point “A”).

#### Spine MRI

Intervertebral disc composition [48], vertebral fat fraction [49] and paraspinal muscle size and composition [50] will be assessed using a 3T MRI scanner with the following sequences: 1) T2 sag spin-echo multi-echo, 2) Sag DIXON, 3) Axial DIXON, 4) Axial PD and 5) T2-weighted Sag. Radiological assessment will also be performed for the presence of radiological evidence of degenerative changes: facet joint osteoarthritis, pars defects and spondylolisthesis, disc herniation, spinal stenosis, osteophytes, Modic changes and disc degeneration (Pfirrmann grade ≥3; [51]). These outcomes are known to be affected in non-specific LBP [52–54].

#### Tactile spatial acuity assessment

Following the MRI examinations, the two-point discrimination threshold (2PDT) at the lumbar region is to be determined with each test person according to established procedures [55, 56]. In this examination, a test instrument with seven tips separated by various defined distances, or a test instrument with only a single tip, is applied to the subject’s skin for approximately 1 second. The test person is instructed to say whether he or she feels "one" or "two" measuring instruments. Each test instrument is tested 8 times in random order. The 2PDT is defined as the distance of the peaks at which participants answered "two" 50% of the time.

#### Pressure pain thresho1lds

Pressure-pain thresholds (PPT) will be assessed using a digital algometer at the lumbar paraspinals (local hyperalgesia) and at the elbow and calf (for widespread hyperalgesia) using standard protocols [57, 58]. Changes in PPTs indicates local and/or widespread pain sensitivity in patients with non-specific LBP [59]. Manual pressure will be applied using a 10mm‐diameter rod at a rate of 10 N/cm^2^. Subjects will be instructed to say ‘pain’ or ‘stop’ at the point pressure turns to pain. The protocol will consist of two trials at each site (left, right, left, right) with 20s of rest between each trial. Order for calves, arms, back will be randomised. The average data of the two trials at each site will be recorded.

#### Temporal summation of pain

Temporal summation of pain (TS-Pain) [60] will be assessed as per standard protocol [61, 62]. Ten consecutive pulses of the algometer will be applied to the same locations as PPT testing. Pressure of each pulse will be increased at a rate of 2kg/s and a frequency of 1 Hz, until reaching the previously determined PPT and maintained for one second. Participants will rate their perceived pain intensity during the first, fifth and tenth pulse using a verbal numeric rating scale from zero to 10 [63]. TS-Pain is established when the verbal numeric rating scale scores increase from the first to the fifth, and from the fifth to the tenth pulse. The TS-Pain magnitude will be expressed as the first score subtracted from the last, with higher scores indicating more efficacious transmission of nociception to the brain.

#### Pain related evoked potentials (PREPs)

Pain related evoked potentials (PREPs) will be assessed using noxious laser heat stimulation of the pain-affected back and a pain free remote area (abdomen) [64, 65]. By means of a neodymium-doped-yttrium aluminium–perovskite (Nd:YAP) laser with a wavelength of 1,340 nm, a short thermal pulse (1–100 ms) will be applied to intact skin, ensuring synchronous activation of Aδ- and C-thermal nociceptors [65, 66]. To obtain reproducible cortical responses, it is important to apply a suprathreshold pain stimulus intensity that is usually less unpleasant than an electrical stimulus used in somatosensory evoked potential (SEP) recordings [65]. Determination of the stimulus intensity will follow standardized approaches reported elsewhere [67, 68]. The stimulus is often likened to a mild burning or stinging sensation but due to its deeper skin penetration with lower superficial skin heating, the Nd:YAP laser causes only marginally visible skin lesions [65]. Cortical responses will be recorded according to the international 10–20 system [69] over the vertex (Cz) and an earlobe reference [64, 70]. To ascertain the integrity of the complete somatosensory lemniscal conduction pathway, the measurement of tibial nerve SEPs will be added to the PREP protocol. As normative data for this sensory nerve of the lower limb already exist [71], data from the current study population will be comparable to already published data [72] With this additional measurement, potentially hidden abnormalities regarding the conduction velocity of the evoked potentials, which could potentially influence the results from the PREP protocol, could be detected.

#### Cognitive-emotional factors

We will use the standardised and validated Patient-Reported Outcomes Measurement Information System (PROMIS) [73] questionnaires to measure anxiety, depression, cognitive function, self-efficacy, satisfaction social roles and activities, social isolation and social and instrumental support; all known to be impacted in non-specific LBP [74]. As a German version of the PROMIS questionnaire for emotional and instrumental support is not available, a validated German questionnaire [75] will be used for this outcome.

#### Additional musculoskeletal pain locations and cardiovascular risk factors

The presence of other musculoskeletal disease is a known risk factor for non-specific LBP [76] and will be assessed using the Nordic Musculoskeletal Questionnaire [77] which asks for self-report presence of pain in different body regions. Known cardiovascular risk factors for disc herniation (known diabetes, hypertension, high cholesterol and parent who had suffered a myocardial infarction before age 60) will be captured as yes/no response on questionnaires [78].

#### Central sensitisation inventory questionnaire

The Central Sensitisation Inventory questionnaire will assess central sensitisation [79] as a measure of central nervous system hypersensitivity. This tool may be a potential surrogate measure of more time and personnel-costly pain threshold and temporal summation of pain measures. A score of >40 on Part A of the inventory indicates central sensitisation.

#### Demographic and standard outcome measures

We will collect age, occupation (categories per an established taxonomy [80]), weight, height, BMI, smoking history (per the DKFZ-Lymphomstudie [81]), pain duration, pain intensity (visual analogue scale), Oswestry Disability Index, short form of the international physical activity questionnaire [82], Tampa scale for kinesiophobia [83], fear avoidance belief questionnaire [84], current prescription medication, trunk muscle strength [85] and trunk muscle endurance [86]. These measures will characterize the population but not be included in sub-grouping.

### Bias

Project staff conducting the participant testing will be blinded to group status. Should the volunteer reveal their pain status to the operators, the protocol deviation will be noted. Questionnaires will be completed via SoSci (https://www.soscisurvey.de/) and inaccessible to the testers. Data will be encoded by a unique, random, four-digit participant code to blind all steps of analysis.

### Study size

For sub-grouping analyses using machine learning methods, the approach of Foreman [87, 88] to calculate the sample size is used: 2^k^ (where k = number of variables) is minimum sample size and 5*2^k^ is the most conservative sample size. The UK Biobank data study [31, 89] provided only a sub-set of the required parameters, thus the impact of the additional measures in our proposed study (e.g. spine MRI) on sub-grouping is unclear. In this preliminary work [31], less than seven variables were required to characterise a LBP collective, with two specific variables being most important for defining sub-groups. Thus, we argue that using up to 8 variables (2^8^ = 256) across spinal tissue, nervous system and psychosocial domains will likely be sufficient to detect the largest and clinically relevant sub-groups. Therefore, we conservatively target a sample of 300 people with non-specific LBP and 100 controls. This will enable valid subgrouping/classification of patients with non-specific LBP with up to 8 variables in the primary analysis, with additional power for sub-domain analyses with less variables as required. The choice of a 3:1 ratio LBP-to-control was pragmatic for project feasibility and because the focus of our project is on sub-grouping in LBP rather than in pain-free controls.

### Data handling and processing

To promote data quality, questionnaire data entered by the participant and data entered by study staff (e.g. body mass, endurance times) during testing will be collected via SoSci (https://www.soscisurvey.de/). Monthly back-ups of these data from SoSci will be performed during the data collection process onto the servers of the Hochschule für Gesundheit or the Hochschulnetz NRW (“sciebo”). Any events or protocol deviations during testing will be noted in free-text format in the same system. Where feasible, automatic range checking will be implemented during data entry onto SoSci and will be implemented as part of data processing. Where team staff members are not priorly experienced in the outcome measures, training will be provided by experienced team members and/or collaborators along with standard operating procedure documentation. As part of standard practice, MR image data is checked visually by the MR-operator and investigator team member during scanning and scans repeated once if, for example, movement or other artefacts impact the images collected. MR images are measured manually by trained staff using established protocols [48, 49, 90] to obtain the numerical outcomes of interest. The remaining numerical data (e.g. objective physical activity measures, strength, endurance, body mass) will be collected per established standard protocols. A tabulated list of the outcomes is contained in Table 1.

For the data-science approaches implemented, complete data is required. Therefore, missing data will be imputed. The exact method of data imputation will be determined in consultation with a biostatistician once the degree of missingness is known and whether data are “missing-at-random” or not. We anticipate the missingness will be small, as we are performing a cross-sectional study and not a test-retest design, such as in a randomised controlled trial where dropouts inevitably occur. Additional sensitivity analyses performed to assess impact on the findings.

### Analytical and statistical methods

**Fig 1:**
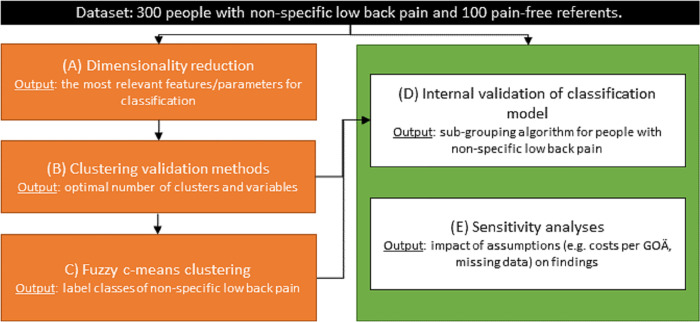
Approach to artificial intelligence tool implementation. GOÄ: Gebührenordnung für Ärzte (German medical cost database).

The following approach, based on our preliminary work [31, 47], will be used (see also Fig 1).

- Dimensionality reduction will be performed to identify the most relevant variables (of those listed; Table 1) for subgrouping and classification. Given known challenges with data-analytic variable weighting and multidimensional data (e.g. scaling each variable to 0–1) [31], our first step in dimensionality reduction will focus on traditional statistical methods including paired t-tests, analysis of covariance and multicollinearity assessment through correlation analysis [31]. Following this, pending the number of important variables (e.g. if more than 8 variables remain) we will consider variable weighting using a decision tree classifier and targeted projection pursuit with artificial neural networks [91]. The outputs will be used to select the most relevant variables distinguishing non-specific LBP from controls (2-class model).
- Following determination of the most important variables, pain-free controls will be removed from the data for the following clustering steps. Cluster validation methods [92], including Calinski-Harabasz, Davies-Bouldin and Sillouette values, will be used to determine the optimal number of clusters and variables for the remaining non-specific LBP data. Given the potential for high dimensionality introducing noise and influencing clustering, we will evaluate the outputs of clustering using all outcomes and within individual domains (e.g. in preliminary work [31, 47], psychological and brain MRI variables correlated more highly with each other within their domains than between variable domains). The output of this step will allow us to determine the optimal number of clusters and variables.
- Based on the information in step B, we will apply fuzzy c-means clustering model [31] of non-specific LBP on either the primary clustering or domain specific clustering. The clusters derived in this step will be used to label classes of non-specific LBP.
- Outputs from steps B and C in terms of class labels will be used to evaluate non-specific LBP classification. We will apply Support Vector Machine, Naïve Bayes, k-Nearest Neighbour and Random Forest binary/multiclass classifiers as appropriate for the data. Given we are focusing on internal (i.e. on the current dataset, rather than a second newly collected dataset from a different research centre or project) validation of a classification model, validation of the model will be done via k-fold cross validation, as random data-splitting is not generally recommended for internal model validation [93–96]. The output of this will be a sub-grouping tool for people with non-specific LBP assuming availability of all data and tests and no consideration of test cost.
- We will consider the following sensitivity analyses:

1. we will evaluate random data-splitting and repeat steps B and C to determine if the clustering outputs hold across random samples;
2. we will apply steps B to E within each domain (e.g. brain MRI, psychosocial variables, spine MRI) as required;
3. repetition of analysis only with participants without any imputed data; to examine the potential impact of missing data.
4. consideration of impact of secondary variables (see bottom panel of Table 1) on sub-grouping.
5. the methods of step (D) will be reapplied under constrained cost assumptions, considering financial-cost, time, and patient burden of obtaining each clinical outcome. Financial costs will be according to current rates according to the Gebührenordnung für Ärzte; time will be according to clinical measure and patient burden will be according to an ordinal scale. The output will be a model for most effective sub-group identification for people with non-specific LBP respecting cost/time/burden constraints.
6. consider the findings of sub-grouping when patients with or without radiological evidence of degenerative changes (see Quantitative variables and data collection > Spine MRI) are included.

### Confidentiality and data management

Data protection regulations for scientific investigations of the state of North Rhine-Westphalia (§28 DSG-NRW) and the General Data Protection Regulation (Art. 89 DSGVO) will be observed. During measurements with participants in the study, image (DICOM format) and tabulated numerical and string data will be generated (“study data”) in electronic form. Personal data (name, date of birth) will be recorded on a written informed consent form. Each participant will be assigned a random code (e.g. PREDICT34621). This code connects the consent form and the study data. We expect to that up a maximum of 30 GB of data will be generated in the study. Personal data on the consent form will not be saved with the data collected during the study. The consent form containing the personal data and the participant code will be stored separately from the study data at the Hochschule für Gesundheit and will be destroyed by the project management after the study has been completed. The study data is thus pseudonymised until the consent form is destroyed. After destroying the consent form, the study data are anonymized. Participation in the study is voluntary and participants can withdraw at any time and request their data be deleted. After anonymization, however, it is no longer possible to revoke participation since the data can then no longer be assigned to a single person. All study data will be stored electronically in a pseudonymised (later anonymous) form, protected by a password, on the servers of the Hochschule für Gesundheit, Universitätsklinikum Bergmannsheil and/or the Hochschulnetz NRW (“sciebo”). Data obtained at Bergmannsheil Bochum will be stored on site on local servers. In the interest of good scientific practice, the study data will be permanently stored in an anonymous form in online register databases after the study has been completed. Anonymized participant-level tabulated data will be made available permanently on a GDPR-conform data repository, such as www.osf.io and/or as supplemental data with published manuscripts. As a rule, anonymized participant-level tabulated data will be transferred to online register databases when the results are published in specialist journals, but at the earliest after data collection for the study has been completed. This will enable free re-use of the collected data. For MR (DICOM format) image data, access to the image data and re-use will be permitted upon written agreement with the Hochschule für Gesundheit.

### Dissemination policy

Findings from the outcomes of this trial will be reported in journal articles and potentially PhD theses, which will include results regardless of the direction or magnitude of the effect. The results will also be presented at leading national and international conferences, clinical forums and to other relevant health professionals and stakeholders, as well as to the participants. All participants will be able to elect to receive a brief report of their study results via email at the completion of the study. Participants may also receive a copy of their MRI data. If the participant expresses interest in the study results, they can request to have a copy of published articles sent to them.

Consistent with our current practice, the ICJME authorship guidelines [97] will be followed in defining author and non-authors and author roles will be follow the CRediT taxonomy [98, 99]. We will publish the full protocol in a peer-reviewed journal. Anonymized participant-level data and statistical code will be made available on a data repository, such as www.osf.io and/or as supplemental data with published manuscripts.

## Discussion

Based on our recent systematic review [23], our project will be the first larger scale study to assess multiple dimensions of pain contributors with a view to detecting clinically relevant sub-groups in non-specific LBP. We anticipate the production of a comprehensive, multidimensional dataset including imaging of the lumbar spine and the brain, assessment of pain processing, and emotional and psychosocial determinants of pain. This dataset will position us to test the hypothesis that patients with non-specific LBP can be grouped in clusters with common features.

Currently non-specific LBP is viewed as one large all-encompassing diagnostic entity. In our view, the results of the project, if sub-groups do indeed exist, will result in a substantial departure from the current paradigm by taking a fresh, rational perspective to optimise the screening of NSCLBP, which will guide more focused and tailored management. Importantly, should no relevant sub-groups be detected, this will also be a boon for current efforts, in that this will reinforce the need to implement current (generic) approaches, such as reducing medical imaging, for the management of non-specific LBP.

It is relevant to consider some potential limitations of the study. Firstly, we are conducting a prospective cross-sectional study. This means, that if sub-groups are detected, further prospective cohort and/or interventional studies will need to be conducted to, for example, examine the efficacy of interventions targeted at the individual sub-groups. Nonetheless, the current work is a necessary step along this path. Secondly, the necessary sample size to detect a given sub-group is always unknown. As described in the ‘study size’ section, our sample size will enable detection of sub-groups with up to six outcome per sub-group. We argue that 8 outcomes per sub-group of the outcomes collected will be sufficient to detect the largest clinically relevant sub-groups. Nonetheless, there may be smaller sub-groups for which more parameters are needed to detect them in non-specific LBP. However, we question whether an overall reduction in the collective burden of disease is possible by targeting such (small) sub-groups. Thirdly, whilst we aim to assess key known domains in the multidimensional contributors in LBP, we do not assess every known possible variable. We observed in our preliminary work [31, 47, 89] that parameters within a given domain often co-correlate. Further, there is no one single parameter that “explains” non-specific LBP. Therefore, we argue that additional parameters may have further explanatory power, but that it is unlikely that they would make a decisive different in the final clinical approach.

## Supporting information

S1 Table STROBE checklist. (DOCX)

S2 Table TRIPOD checklist. (DOCX)
