# Supplementary material for: Evidence- and data-driven classification of low back pain via artificial intelligence: Protocol of the PREDICT-LBP study
Source: PLoS One. 2023 Aug 21;18(8):e0282346. doi: 10.1371/journal.pone.0282346 (PMC10441794; doi:10.1371/journal.pone.0282346)
Supplement: S2 Table — (DOCX) [file pone.0282346.s002.docx]

**S2 Table**. TRIPOD Checklist.

| **Section/Topic Item Checklist Item Page** | | | |
| --- | --- | --- | --- |
| **Title and abstract** | | | |
| Title | 1 | Identify the study as developing and/or validating a multivariable prediction model, the target population, and the outcome to be predicted. | 1 |
| Abstract | 2 | Provide a summary of objectives, study design, setting, participants, sample size, predictors, outcome, statistical analysis, results, and conclusions. | 3 |
| **Introduction** | | | |
| Background and objectives | 3a | Explain the medical context (including whether diagnostic or prognostic) and rationale for developing or validating the multivariable prediction model, including references to existing models. | 4 |
|  | 3b | Specify the objectives, including whether the study describes the development or validation of the model or both. | 5 |
| **Methods** | | | |
| Source of data | 4a | Describe the study design or source of data(e.g., randomized trial,cohort, or  registry data), separately for the development and validation data sets, if applicable. | 7 |
|  | 4b | Specify the key study dates, including start of accrual; end of accrual; and, if applicable, end of follow-up. | 7 |
| Participants | 5a | Specify key elements of the study setting (e.g., primary care, secondary care, general population) including number and location of centres. | 7-8 |
|  | 5b | Describe eligibility criteria for participants. | 8-10 |
|  | 5c | Give details of treatments received, if relevant. | NA |
| Outcome | 6a | Clearly define the outcome that is predicted by the prediction model, including how and when assessed. | 17-19 |
|  | 6b | Report any actions to blind assessment of the outcome to be predicted. | 16 |
| Predictors | 7a | Clearly define all predictors used in developing or validating the multivariable  prediction model, including how and when they were measured. | 10-15 |
|  | 7b | Report any actions to blind assessment of predictors for the outcome and other predictors. | 16 |
| Sample size | 8 | Explain how the study size was arrived at. | 16 |
| Missing data | 9 | Describe how missing data were handled (e.g., complete-case analysis, single imputation, multiple imputation) with details of any imputation method. | 17 |
| Statistical analysis methods | 10a | Describe how predictors were handled in the analyses. | 18-19 |
|  | 10b | Specify type of model, all model-building procedures (including any predictor selection), and method for internal validation. | 18-19 |
|  | 10d | Specify all measures used to assess model performance and, if relevant, to compare multiple models. | 18-19 |
| Risk groups | 11 | Provide details on how risk groups were created, if done. | 18-19 |
| **Results** | | | |
| Participants | 13a | Describe the flow of participants through the study, including the number of participants with and without the outcome and, if applicable, a summary of the follow-up time. A diagram may be helpful. | NA |
|  | 13b | Describe the characteristics of the participants (basic demographics, clinical features, available predictors), including the number of participants with missing  data for predictors and outcome. | NA |
| Model development | 14a | Specify the numberof participants and outcome events in each analysis. | NA |
|  | 14b | If done, report the unadjusted association between each candidate predictor and outcome. | NA |
| Model specification | 15a | Present the full prediction model to allow predictions for individuals (i.e., all regression coefficients, and model intercept or baseline survival at a given time  point). | NA |
|  | 15b | Explain how to the use the prediction model. | NA |
| Model performance | 16 | Report performance measures (with CIs) for the prediction model. | NA |
| **Discussion** | | | |
| Limitations | 18 | Discuss any limitations of the study (such as nonrepresentative sample, few events  per predictor, missing data). | 22-23 |
| Interpretation | 19b | Give an overall interpretation of the results, considering objectives, limitations, and  results from similar studies, and other relevant evidence. | 22 |
| Implications | 20 | Discuss the potential clinical use of the model and implications for future research. | 22 |
| **Other information** | | | |
| Supplementary information | 21 | Provide information about the availability of supplementary resources, such as study protocol, Web calculator, and data sets. | 2 |
| Funding | 22 | Give the source of funding and therole of the funders for the presentstudy. | 2, 24 |

**S3 Table.** Author contributions (CRediT table)

| **Term** | **Who?** | **Definition** |
| --- | --- | --- |
| Conceptualization | DLB, ST, MT, EE-K, LS, BB, TS, SeS, HJW, MA, GT, KE, BF, JVO, CTM, PJO, SB | Ideas; formulation or evolution of overarching research goals and aims |
| Methodology | DLB, ST, MT, EE-K, LS, BB, TS, SeS, HJW, MA, GT, KE, BF, JVO, CTM, PJO, SB | Development or design of methodology; creation of models |
| Software | NA (protocol manuscript) | Programming, software development; designing computer programs; implementation of the computer code and supporting algorithms; testing of existing code components |
| Validation | NA (protocol manuscript) | Verification, whether as a part of the activity or separate, of the overall replication/ reproducibility of results/experiments and other research outputs |
| Formal analysis | NA (protocol manuscript) | Application of statistical, mathematical, computational, or other formal techniques to analyze or synthesize study data |
| Investigation | NA (protocol manuscript) | Conducting a research and investigation process, specifically performing the experiments, or data/evidence collection |
| Resources | DLB, MT, EE-K, LS, BB, TS, SeS, HJW, MA | Provision of study materials, reagents, materials, patients, laboratory samples, animals, instrumentation, computing resources, or other analysis tools |
| Data Curation | NA (protocol manuscript) | Management activities to annotate (produce metadata), scrub data and maintain research data (including software code, where it is necessary for interpreting the data itself) for initial use and later reuse |
| Writing - Original Draft | DLB, RD, SK, ST | Preparation, creation and/or presentation of the published work, specifically writing the initial draft (including substantive translation) |
| Writing - Review & Editing | DLB, ST, MT, EE-K, LS, BB, TS, SeS, HJW, MA, GT, KE, BF, JVO, CTM, PJO, SB, RD, SK | Preparation, creation and/or presentation of the published work by those from the original research group, specifically critical review, commentary or revision – including pre-or postpublication stages |
| Visualization | NA (protocol manuscript) | Preparation, creation and/or presentation of the published work, specifically visualization/ data presentation |
| Supervision | DLB | Oversight and leadership responsibility for the research activity planning and execution, including mentorship external to the core team |
| Project administration | DLB | Management and coordination responsibility for the research activity planning and execution |
| Funding acquisition | DLB, ST, MT, EE-K, LS, BB, TS, SeS, HJW, MA, GT, KE, BF, JVO, CTM, PJO, SB | Acquisition of the financial support for the project leading to this publication |
