# Supplementary material for: Evidence- and data-driven classification of low back pain via artificial intelligence: Protocol of the PREDICT-LBP study
Source: PLoS One. 2023 Aug 21;18(8):e0282346. doi: 10.1371/journal.pone.0282346 (PMC10441794; doi:10.1371/journal.pone.0282346)
Supplement: Peer review history

# Editor report: Decision Letter 0

**DOI:** 10.1371/journal.pone.0282346.r001

**Authors:** James Mockridge

14 Feb 2023

Evidence- and data-driven classification of low back pain via artificial intelligence: protocol of the PREDICT-LBP study

PONE-D-22-31941

Dear Dr. Belavy,

We’re pleased to inform you that your manuscript has been judged scientifically suitable for publication and will be formally accepted for publication once it meets all outstanding technical requirements.

Within one week, you’ll receive an e-mail detailing the required amendments. When these have been addressed, you’ll receive a formal acceptance letter and your manuscript will be scheduled for publication.

An invoice for payment will follow shortly after the formal acceptance. To ensure an efficient process, please log into Editorial Manager at http://www.editorialmanager.com/pone/, click the 'Update My Information' link at the top of the page, and double check that your user information is up-to-date. If you have any billing related questions, please contact our Author Billing department directly at authorbilling@plos.org.

If your institution or institutions have a press office, please notify them about your upcoming paper to help maximize its impact. If they’ll be preparing press materials, please inform our press team as soon as possible -- no later than 48 hours after receiving the formal acceptance. Your manuscript will remain under strict press embargo until 2 pm Eastern Time on the date of publication. For more information, please contact onepress@plos.org.

Kind regards,

James Mockridge

Staff Editor

PLOS ONE

Journal Requirements:

​

1. Thank you for stating the following in the Competing Interests section: 

DLB, ST, MT, LS, SeS, HJW, MA, GT, KE, BF, JVO, CTM, PJO, SB, RD, SK declare no conflicts of interest. 

BB: declares no conflicts of interest relevant to the current work, but has previously received research support, consultancy fees and/or honoraria from AbbVie, Amgen, Biogen, GE/Lunar, Janssen, Galapagos, Gilead, Medimaps, MSD, Sanofi Genzyme, Theramex, UCB. 

TS: declares prior consulting fees from Johnson & Johnson, Amgen, Kaia Health Software, SpineArt, Implantcast and speaking and  travel arrangements from Johnson & Johnson, ICOTEC, Nuvasive, Ulrichmedical, Silony, Amgen, Kaia Health Software.

EE-K has received fees from painCert GmbH, Casquar GmbH and Omega Pharma GmbH, outside the submitted work.

Please confirm that this does not alter your adherence to all PLOS ONE policies on sharing data and materials, by including the following statement: "This does not alter our adherence to  PLOS ONE policies on sharing data and materials.” (as detailed online in our guide for authors http://journals.plos.org/plosone/s/competing-interests). If there are restrictions on sharing of data and/or materials, please state these. Please note that we cannot proceed with consideration of your article until this information has been declared. 

Please respond by return email with your amended Competing Interests Statement and we will change the online submission form on your behalf.

# Editor report: Acceptance letter

**DOI:** 10.1371/journal.pone.0282346.r002

**Authors:** James Mockridge

28 Mar 2023

PONE-D-22-31941 

Evidence- and data-driven classification of low back pain via artificial intelligence: protocol of the PREDICT-LBP study 

Dear Dr. Belavy:

I'm pleased to inform you that your manuscript has been deemed suitable for publication in PLOS ONE. Congratulations! Your manuscript is now with our production department. 

If your institution or institutions have a press office, please let them know about your upcoming paper now to help maximize its impact. If they'll be preparing press materials, please inform our press team within the next 48 hours. Your manuscript will remain under strict press embargo until 2 pm Eastern Time on the date of publication. For more information please contact onepress@plos.org.

If we can help with anything else, please email us at plosone@plos.org. 

Thank you for submitting your work to PLOS ONE and supporting open access. 

Kind regards, 

PLOS ONE Editorial Office Staff

on behalf of

Dr James Mockridge 

Staff Editor

PLOS ONE
